# OA12 Grimey granulomas of sarcoidosis

**DOI:** 10.1093/rap/rkad070.012

**Published:** 2023-09-27

**Authors:** Deepak Nagra, Maryam Adas, Clara Watkins, Rosaria Salerno, Louise Ameyaw-Kyeremeh, Christopher Baldwin, Mark Russell, Katie Bechman, Corrine Byrne, James Galloway

**Affiliations:** King's College Hospital, London, United Kingdom; Centre for Rheumatic Disease, King's College London, London, United Kingdom; Centre for Rheumatic Disease, King's College London, London, United Kingdom; King's College Hospital, London, United Kingdom; King's College Hospital, London, United Kingdom; King's College Hospital, London, United Kingdom; King's College Hospital, London, United Kingdom; King's College Hospital, London, United Kingdom; Centre for Rheumatic Disease, King's College London, London, United Kingdom; King's College Hospital, London, United Kingdom; Centre for Rheumatic Disease, King's College London, London, United Kingdom; King's College Hospital, London, United Kingdom; King's College Hospital, London, United Kingdom; Centre for Rheumatic Disease, King's College London, London, United Kingdom

## Abstract

**Introduction:**

Sarcoidosis affects approximately 1% of the population. There are two situations where it came become life threatening: seizures secondary to neuro-sarcoidosis and arrhythmias due to cardiac infiltration. Currently there are limited licenced therapies for the treatment of sarcoidosis. We describe a case of cutaneous and neuro-sarcoidosis who developed subsequent multi-system sarcoidosis.

**Case description:**

A previously fit and well 50-year-old presented to his local hospital with tonic clonic seizures. He worked as a mortgage broker and never smoked. There was no pro-drome and on examination it was noted he had erythema nodosum on his shins. He was investigated with a CT head and subsequent MRI demonstrating enhancement of his leptomeninges, mid brain parenchyma, hypothalamus and pituitary gland. A lumbar puncture was performed with an elevated protein level of 934g with a normal glucose and lymphocyte level. The CSF was negative for TB. A CT of his chest was performed demonstrating bilateral hilar lymphadenopathy and subsequent histology of these lymph nodes demonstrated non-necrotising, non-caseating epithelioid granulomas. A diagnosis of sarcoidosis was made and he was commenced on mycophenolate. His disease remained quiescent for 5 years before the eruption of further skin lesions. He developed multiple subdermal nodules on his fingers, wrists, elbows which were biopsied demonstrating cutaneous sarcoidosis. Neurological examination demonstrated brisk lower limb reflexes with upgoing plantars. He was commenced on a tapering course of prednisolone and an evaluation for extra-cutaneous disease was made. An IL2Ra was 4719ng/L (423-1843) with a normal ACE. Pituitary axis testing revealed a slightly raised prolactin (326 mIU/L). A CT-PET was performed demonstrating extensive metabolic activity in his skin, central nervous system, liver, spleen, salivary glands, skeletal uptake in both humeral heads alongside myocardial uptake. An urgent cardiac MRI was performed demonstrating active myocardial disease. An ECG did not demonstrate any conduction abnormalities. A diagnosis of multi-system sarcoidosis was made warranting an urgent MDT discussion given the progression of his disease.

**Discussion:**

In this particular case, it had been assumed that his sarcoidosis was inactive until the emergence of the skin lesions. It was the skin lesions that lead to an assessment for multi-system sarcoidosis. It is common for the serum ACE level to be normal, as seen in up to 50% of cases and it was the Il2Ra elevation that prompted the CT-PET request. Given the extent and progression of disease despite treatment with mycophenolate, this gentlemen was given a tapering regime of prednisolone (starting at 20mg) and his mycophenolate was switched to methotrexate which has shown to have superior efficacy to mycophenolate for sarcoidosis. Furthermore, infliximab at a dose of 5mg/kg was commenced shortly after to control the increasing CNS lesions. Infliximab is a well recognised treatment for sarcoidosis; however, due to limited clinical trial data, it remains unlicenced for use in sarcoidosis with the exception of neuro-sarcoidosis (following the failure of DMARD therapy). To date, the patient remains well with no further worsening in his disease.

**Key learning points:**

Sarcoidosis is a clinical entity with no one specific test. The diagnosis should be constructed using multiple parameters including radiographic findings, biochemical and histological proof. We advocate that suspected cases of sarcoidosis should have a thorough assessment and discussion in the MDT. When isolated skin disease is suspected or confirmed, baseline testing for extra-cutaneous involvement should be sent including NT-proBNP, troponin I and T, creatinine kinase, a full pituitary profile, ACE and cytokine assessment for Il2Ra, amongst other routine blood tests. An ECG is of paramount importance as arrhythmias are common cardiac features of sarcoidosis. Should there be ongoing suspicion of multiple organ involvement, a CT-PET should be performed at baseline. Isolated lymph node disease does not commonly warrant treatment; however, this cohort of patients should be closely monitored. The management of sarcoidosis in the acute setting is commonly prednisolone, but steroid sparing therapies should be considered in patients needing recurrent, prolonged courses of corticosteroids or those with evidence of critical organ involvement such as CNS, cardiac or lung disease. Life threatening disease or disease refractory to DMARD therapy should be considered for anti-TNF therapy with infliximab or adalimumab. More recently, tofacitinib has been promising as a treatment for sarcoidosis with clinical trials in Yale and Portland. Newer therapies in development include the GM-CSF inhibitor namilumab, and efzofitimod, a neuropilin 2 inhibitor.

